# Serum under-carboxylated osteocalcin levels in women with polycystic ovary syndrome: weight-dependent relationships with endocrine and metabolic traits

**DOI:** 10.1186/1757-2215-6-4

**Published:** 2013-01-22

**Authors:** Carmen E Pepene

**Affiliations:** 1 Endocrinology Chair, 6th Medical Sciences Department, “Iuliu Hatieganu” University of Medicine and Pharmacy, 3-5 Louis Pasteur, Cluj-Napoca, 400349, Romania; 2 Endocrinology Clinic, Cluj County Emergency Hospital, Cluj-Napoca, Romania

**Keywords:** Polycystic ovary syndrome, Osteocalcin, Testosterone, Insulin, Anovulation, Bone turnover, Obesity

## Abstract

**Background:**

Under-carboxylated osteocalcin (ucOC), the precursor substrate of bone biomarker OC is a potent regulator of energy metabolism by promoting insulin production and adiponectin synthesis and decreasing fat stores. The aim of the present study was to point out the potential role of ucOC in the physiopathology of polycystic ovary syndrome (PCOS), a common disorder defined by the constellation of anovulation, insulinresistance, hyperinsulinemia, obesity and androgen excess.

**Methods:**

In this prospective case–control investigation, 78 young premenopausal women, *i.e.* 52 PCOS patients and 26 age- and body mass index (BMI)-matched healthy controls, were successively enrolled. Recruitment of PCOS patients was performed according to Androgen Excess-Polycystic Ovary Syndrome (AE-PCOS) Society 2006 criteria. All study participants were subjected to clinical examination, whole-body composition assessment and measurements of serum ucOC, OC (1-49), glucose and lipids, insulin, total testosterone (TT), estradiol, sex-hormone binding globulin (SHBG), high-sensitivity C-reactive protein (Hs-CRP) and β-CrossLaps.

**Results:**

BMI-stratified multivariate analysis revealed significantly higher ucOC levels in PCOS vs. controls in lean (p = 0.001) but not overweight and obese study participants (p = 0.456). Notably, a positive correlation between ucOC and TT (p = 0.018), calculated free testosterone (cFT, p = 0.028) and serum insulin (p = 0.036), respectively, was found to be confined to the lean analysis subgroup. Furthermore, in stepwise multiple regression models, β-CrossLaps and cFT were able to predict 46.71% of serum ucOC variability. (1-43/49)OC failed to be significantly associated to any PCOS trait.

**Conclusions:**

Circulating ucOC concentration is related to key endocrine PCOS characteristics in a weight-dependent manner. Within the bone-pancreas loop, high ucOC may favor insulin release in lean hyperandrogenic women to compensate for impaired insulin sensitivity.

## Background

Osteocalcin (OC), a traditional osteoblast-/odontoblast-secreted biomarker, is synthesized as under-carboxylated precursor molecule and further processed by posttranslational vitamin K-dependent γ-glutamylcarboxylation to deliver the mature, carboxylated bone matrix-bound protein. Apart from reasonable evidence of association to low bone mass [1] and subsequent fracture risk [2], circulating OC in its under-carboxylated form (ucOC) was recently acknowledged as one missing clue in the endocrine cross-talk between the skeleton and energy metabolism. Groundbreaking studies revealed that ucOC may impact glucose metabolism control by stimulating expression of several ß-cell proliferation genes [3] and promoting expression of the insulin gene and increasing insulin production within ß-cells [4]. Hence, ucOC acts as an insulin secretagogue. In addition to that, ucOC may positively control energy metabolism through the release of adiponectin, an insulin-mimetic adipocytokine, from fat cells [3]. In turn, both OC gene expression and OC decarboxylation in osteoblast-like cells are up-regulated by insulin receptor signaling *via* osteoprotegerin (OPG)/ligand of receptor activator of nuclear factor kappa B (RANKL) pathway [5].

Notably, an extracellular/circulating γ-decarboxylase to convert carboxylated OC into ucOC is not described yet. Therefore, it is suggested that the skeleton represents the primary source of ucOC. In fact, serum ucOC results on one hand from osteoblastic cell secretion and on the other hand from decarboxylation of matrix-bound carboxylated OC through resorption of bone. More recently, the adipose tissue was recognized as an OC and ucOC source and ucOC release in the human adipose tissue culture medium was confirmed [6].

Recent studies specifically analyzed the levels of circulating OC gene products in relation to metabolic parameters in human showing significant relationships of OC or ucOC to body mass index (BMI), body fat, glycemic status and HbA1c in adult diabetic [7] and non-diabetic subjects [8,9] even in prospectively planned analyses [10]. Short-term (*i.e.* 3 months) changes in serum ucOC levels in women on anti-fracture therapy for postmenopausal osteoporosis on either anti-catabolic or osteo-anabolic therapy correlate positively to the twelve-months variations in body weight, body fat mass and serum adiponectin, another evidence linking ucOC levels to major metabolic indices [11].

Polycystic ovary syndrome (PCOS) represents the main androgen excess disorder in women of reproductive age, exhibiting polymorph phenotypes and coexistence of obesity and/or insulin resistance in up to 50% of cases [12]. Nevertheless, the intimate mechanism of inappropriate glucose metabolism control in these women remains incompletely defined. Reports focused on androgen hormone administration in lean female-to-male transsexuals suggest that insulin resistance in the hyper-androgenic women cannot be attributed solely to androgen excess despite evidence of visceral fat accumulation [13]. Additionally, both obese and non-obese phenotypes of PCOS women may develop impaired insulin sensitivity [14,15], oxidative stress [16] and even endothelial injury [17-19].

The novel description of OC as a metabolic marker raised the question of its potential implication in the pathogenesis of PCOS. In one previous study, significantly higher carboxylated OC (cOC) concentration was reported in patients with PCOS compared to controls thus suggesting a potential relationship between PCOS status and dynamics of the OC γ-carboxylation process [20]. Moreover, cOC and OC displayed by PCOS patients in that study correlated with several PCOS endocrine and metabolic components. In this prospective and cross-sectional survey, circulating ucOC levels were directly assayed for the first time in PCOS patients using a dedicated two monoclonal antibodies ELISA. The relationships of serum ucOC concentrations to body fat, serum androgens, hyperinsulinism and low-grade inflammation were investigated.

## Methods

Between January 2009 and June 2010, 78 Caucasian women (52 patients with PCOS and 26 healthy controls, aged 24.620 ± 0.688 years) were successively recruited and managed on an outpatient basis at a tertiary endocrine care center. Androgen Excess-Polycystic Ovary Syndrome (AE-PCOS) Society 2006 Task Force diagnostic criteria for PCOS were employed and patients with hirsutism (= or >8 on the Ferriman-Gallwey scoring system) and/or biochemical hyperandrogenism, and oligo-anovulation (history of no more than 8 spontaneous menses in the previous year) and/or polycystic ovaries (>12 follicles 2–9 mm diameter and/or ovarian volume >10 cm^3^ on ultrasound) were enrolled. Subjects with confirmed androgen-secreting tumors, congenital adrenal hyperplasia and Cushing’s syndrome, hyperprolactinemia or thyroid disease as well as current or previous (within 6 months) use of oral contraceptives, metformin, glitazones, statins, anti-androgens, infertility medication or drugs known to affect the carbohydrate-lipid metabolism were excluded. A control group of age- and BMI-matched eugonadal women without clinical and/or biochemical evidence of hyperandrogenism was created and all the aforementioned exclusion criteria were applied to the control group. The study was conducted with the approval of the local ethics committee on clinical investigations and informed consent was obtained from all participants.

Careful physical examination of subjects was done and data on height (cm), weight (kg), the Ferriman-Gallwey score and blood pressure (mm Hg) was recorded in each study participant. The body mass index was calculated as weight (kilograms) divided by height (meters) squared (kg/m^2^). Body mass index (BMI) cutoff between lean and overweight and obese individuals was considered 25 kg/m^2^. Systolic blood pressure (SBP) and diastolic blood pressure (DBP) were measured in the right arm, with the subjects in a seated position. The average of two measurements taken with a mercury sphygmomanometer was used.

Employing the whole-body dual X-ray absorptiometry technique using DPX-NT (GE, Madison, USA) equipment, body composition parameters (*i.e.* total body fat mass and total body fat-free mass) were determined in a fasting state, during the same visit the blood samples were collected. Quality control was ensured by constant calibration using both the phantom provided by the manufacturer and the HOLOGIC 1540 phantom. The coefficient of variance (CV), evaluated at 3% for total body fat mass was determined by serial measurements on 10 patients, each one examined 3 times.

Blood samples were obtained between 08.00 and 10.00 a.m., after overnight fasting and during early follicular phase of a spontaneous or dydrogesterone-induced menstrual cycle. The serum obtained immediately after blood collection was stored at −80°C until the time of assay. Insulin, total testosterone (TT), sex hormone-binding globulin (SHBG), estradiol and high-sensitivity C-reactive protein (Hs-CRP) were all measured using ELISA kits from DRG Instruments, Marburg, Germany. According to Vermeulen’s formula, calculated free testosterone (cFT) was obtained from TT and SHBG serum concentration. As in previous work [19], the cFT cutoff level was defined as 0.028 nmol/l. Serum β-CrossLaps ELISA was purchased from Immunodiagnostic Systems Ltd, Boldon, UK.

Serum OC was quantitatively assayed using the Osteocalcin (1-43/49) ELISA kit from ALPCO Diagnostics, Salem, NH, USA. The lowest detectable limit for OC was 0.31 ng/ml and the mean intra-assay CV was 4.7%. Duplicate serum ucOC concentrations were obtained by a commercial two monoclonal antibodies ELISA from Takara Bio Inc, Otsu, Japan. The mean intra-assay CV of was 4.4%. The kit sensitivity was 0.5 ng/ml.

### Statistical analysis

Data were expressed as mean ± SD. Continuous data were compared using either the two-tailed *t*-test for independent samples or the Mann–Whitney *U*-test, as appropriate. Subgroup analysis of ucOC and OC after stratifying by BMI was considered. Logarithmic transformations were performed as needed to ensure a normal distribution of continuous variables. Associations of OC to several variables were analyzed by univariate regression. The stepwise multiple regression analysis was used to determine which variables predict serum ucOC levels in the study population.

## Results

Main endocrine and metabolic characteristics of study participants and associations with OC and ucOC are depicted in Tables 1 and 2, respectively. A decline in circulating OC forms with age (OC: r = −0.513, p < 0.0001 and ucOC: r = −0.481, p < 0.0001) was evident in the entire cohort of premenopausal women. In addition, bivariate analysis revealed a highly significant inverse association of ucOC to BMI (r = −0.296, p = 0.008) and total body fat mass (r = −0.300, p = 0.007), respectively. Likewise, a significant trend towards lower serum OC with increasing BMI (r = −0.422, p = 0.0001) and fat mass (r = −0.335, p = 0.002) was observed in the entire cohort. Serum β-CrossLaps, a well-documented bone resorption marker, significantly correlated with both OC (r = 0.456, p < 0.0001) and ucOC (r = 0.450, p < 0.0001), respectively, a relationship not confounded by age and BMI for ucOC (p = 0.034 in multivariate analysis).

**Table 1:** Main study parameters assessed in PCOS patients and healthy eugonadal women (n = 78)

	**PCOS (n = 52)**	**Controls (n = 26)**
Age (years)	24.44 ± 5.49	25.96 ± 7.28
BMI (kg/m^2^)	28.79 ± 5.66	27.03 ± 6.04
WHR	0.85 ± 0.06	0.79 ± 0.06**
Total body FM (kg)	32.94 ± 11.58	28.79 ± 11.49
SBP (mm Hg)	108.46 ± 12.46	107.88 ± 12.66
DBP (mm Hg)	72.01 ± 9.96	70.38 ± 9.26
ucOC (ng/ml)	3.11 ± 0.68	2.79 ± 0.84^#^
OC (ng/ml)	13.76 ± 6.58	13.07 ± 6.55
β-CrossLaps (ng/ml)	0.539 ± 0.426	0.528 ± 0.334
TT (nmol/l)	3.04 ± 1.20	1.91 ± 0.80***
cFT (nmol/l)	0.051 ± 0.027	0.020 ± 0.012***
SHBG (nmol/l)	47.58 ± 32.09	88.03 ± 41.19***
Glucose (mg/dl)	85.57 ± 7.90	84.53 ± 7.03
Insulin (μU/ml)	16.56 ± 6.83	12.40 ± 3.81*
HOMA-IR	3.54 ± 1.64	2.60 ± 0.87**
QUICKI	0.322 ± 0.020	0.334 ± 0.017*
Hs-CRP (mg/l)	5.51 ± 3.91	4.04 ± 3.87

Results are presented as mean ± SD and compared by Mann Whitney or two-tailed independent *t*-test, as appropriated. Abbreviations: *BMI*, body mass index, *WHR*, waist-to-hip ratio, *FM*, fat mass, *SBP*, systolic blood pressure, *DBP*, diastolic blood pressure, *ucOC*, under-carboxylated osteocalcin, *OC*, (1-43/49) osteocalcin, *TT*, total testosterone, *cFT*, calculated free testosterone, *SHBG*, sex hormone-binding globulin, *HOMA-IR*, homeostasis model assessment for insulin resistance, *QUICKI*, quantitative insulin sensitivity check index, *Hs-CRP*, high sensitivity C-reactive protein. *p < 0.05, **p < 0.01, ***p < 0.001, ^#^p = 0.070 and p = 0.016 after adjusting for total body fat mass.

**Table 2:** Univariate regression analysis of serum OC with endocrine and metabolic parameters, in PCOS and controls

	**Serum OC**		**Serum ucOC**	
	**PCOS**	**Controls**	**PCOS**	**Controls**
	***β***	***β***	***β***	***β***
Age (years)	−1.159***	−1.535**	−3.648***	−3.045*
BMI (kg/m^2^)	−0.994**	−1.359*	−2.834**	−2.595
Total body FM (g)	−6.173e^−6^ *	−1.067e^−5^	−2.068e^−5^*	−2.550e^−5^
TT (nmol/l)	0.153	−0.173	0.563	−0.351
cFT (nmol/l)	−0.005	0.020	0.230	−0.230
SHBG (nmol/l)	0.078	−0.215	−0.001	0.015
Glucose (mg/dl)	0.282	−0.358	−0.098	1.391
Insulin	−0.198	0.223	−0.650	0.521
HOMA-IR	−0.154	−0.214	−0.552	0.529
QUICKI	1.350	1.951	4.692	−5.120
β-CrossLaps (ng/ml)	0.408***	0.450	1.238**	1.445*
Hs-CRP (mg/l)	−0.068	−0.204*	−0.312	−0.509

*p < 0.05; **p < 0.01; ***p < 0.001.

**Figure 1:**
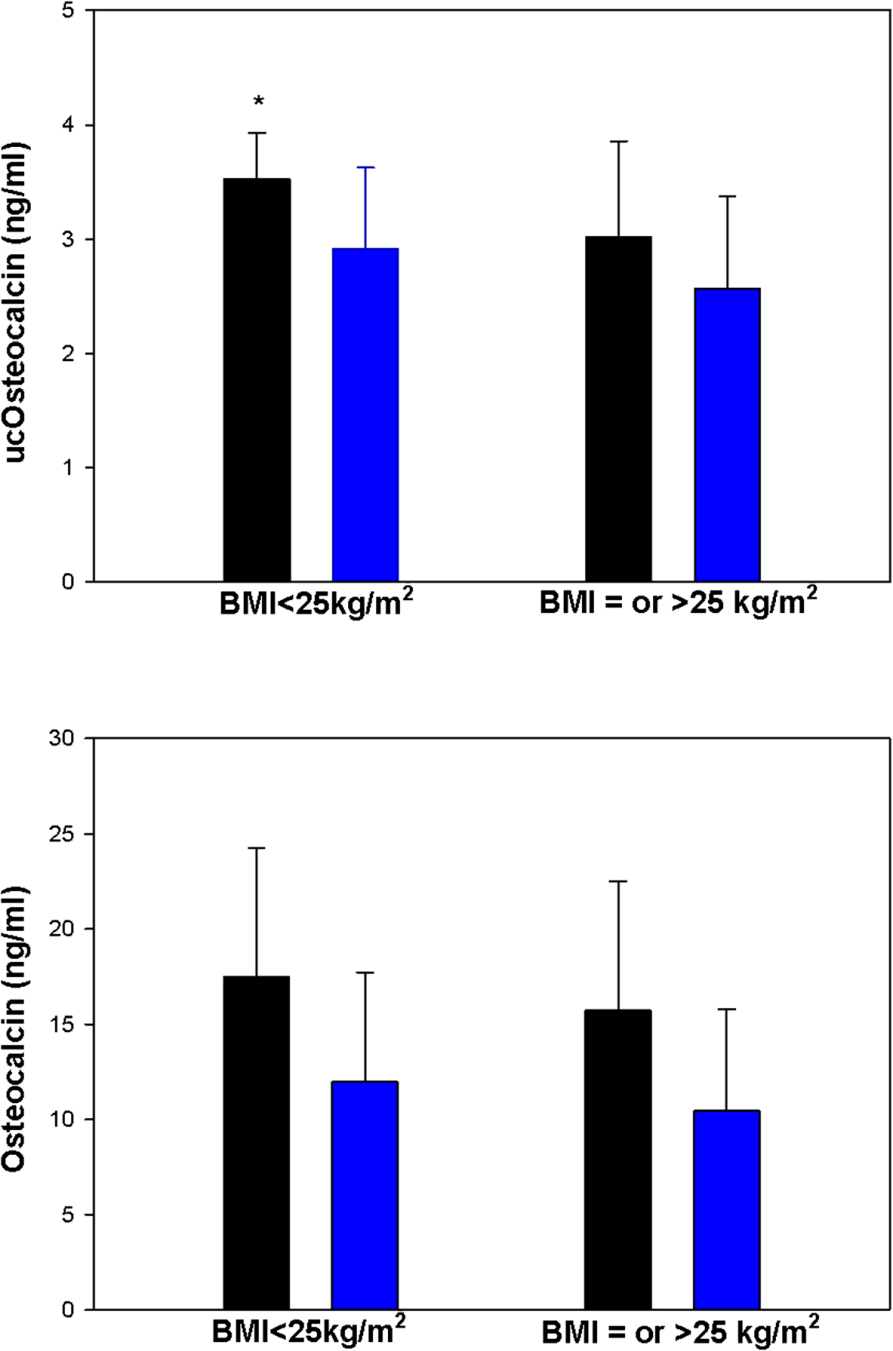
**Body mass index-stratified analysis of osteocalcin (OC) in 52 cases and 26 age-matched controls.** (**upper panel**). Higher under-carboxylated (uc) OC concentration was evidenced in lean (p = 0.037) PCOS patients (n = 17, black bar) vs. controls (n = 13, blue bar) but not overweight and obese (p = 0.490) PCOS patients (n = 35, black bar) vs. controls (n = 13, blue bar), even in multivariate analysis (p = 0.001 in lean and p = 0.456 in overweight and obese). (**lower panel**). Statistical analysis retrieved no differences in serum OC in either lean (p = 0.146) or overweight and obese (p = 0.569) women with PCOS (black bars) and controls (blue bars). Multivariate analysis had no effect on end results (p = 0.535 in lean and p = 0.888 in overweight and obese).

In view of the abovementioned strong relationship between various OC forms and body mass, study subjects were stratified by BMI into lean (BMI < 25 kg/m^2^, n = 30) and overweight and obese (BMI ≥ 25 kg/m^2^, n = 48) subgroups, respectively, and data analysis was done accordingly. As illustrated in Figure 1, lean but not overweight and obese patients with PCOS displayed elevated serum ucOC levels in comparison to controls. Furthermore, the weight-dependent behavior of ucOC strengthened after adjustment for variations in age, body fat mass and bone resorption rate, with a more apparent difference in mean ucOC levels in lean (p = 0.001) but still not significant in overweight and obese (p = 0.456) PCOS patients vs. controls. Nonetheless, BMI-stratified analysis failed to detect any significant differences in circulating OC between PCOS patients and controls in both simple and multivariate analysis (Figure 1).

**Figure 2:**
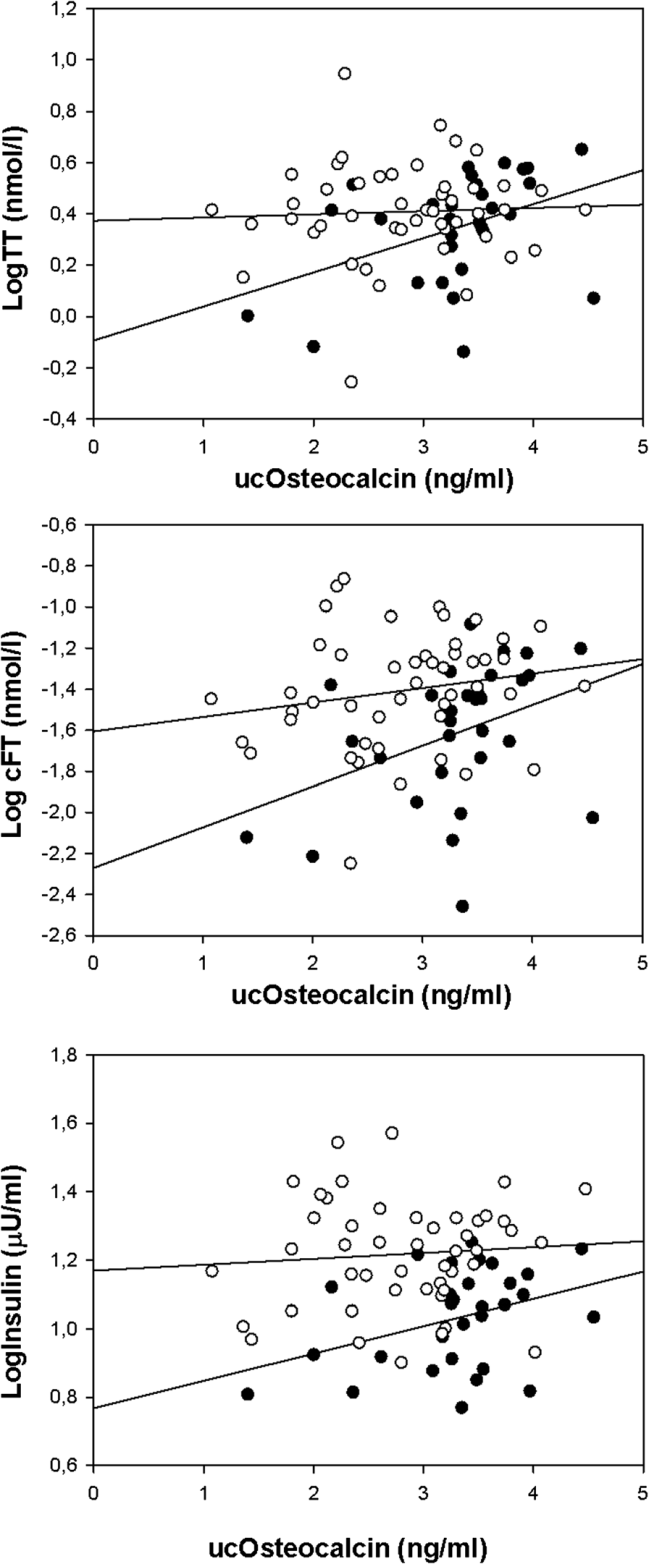
**Scatter plots and regression lines of ucOC association to endocrine traits of study participants (n = 78).** TT = total testosterone, cFT = calculated free testosterone (●) lean women and (○) overweight and obese women. (**upper panel**) (●) r = 0.430, p =0.017 (○) r = 0.049, p = 0.740 (**middle panel**) (●) r = 0.402, p = 0.027 (○) r = 0.181, p = 0.215 (**lower panel**) (●) r = 0.383 p = 0.036 (○) r = 0.081 p = 0.580.

As highlighted in Figure 2, higher serum ucOC was associated in leaner women with higher serum TT (*β* = 0.210, p = 0.018), cFT (*β* = 0.122, p = 0.028) and insulin (*β* = 0.280, p = 0.036) whereas in overweight and obese participants these associations were absent (TT: *β* = 0.042, p = 0.675, FT: *β* = 0.081, p = 0.213, insulin: *β* = 0.079, p = 0.516). In contrast, no relationships with TT, cFT and insulin were evident for OC in either lean (TT: *β* = 0.206, p = 0.232, cFT: *β* = 0.139, p = 0.196 and insulin: *β* = 0.344, p = 0.182) or overweight and obese participants in this study (TT: *β* = 0.068, p = 0.737, cFT: *β* = 0.166, p = 0.209 and insulin: *β* = 0.061, p = 0.802).

In order to find out endocrine and metabolic determinants of serum ucOC, stepwise multiple regression models were established in lean study participants. Therefore, ucOC was introduced as the dependent variable in the model, whereas age, BMI, total body fat mass, β-CrossLaps and PCOS status were considered independent variables. The model (F = 13.885, p < 0.001) showed that β-CrossLaps (p = 0.0006), PCOS status (p = 0.0009) and age (p = 0.017) represented statistically significant determinants of circulating ucOC concentrations. When free testosterone and insulin replaced PCOS status, ucOC was predicted only by β-CrossLaps (p = 0.0002) and cFT (p = 0.003) in the model (F = 13.710, p < 0.001), meaning that 46.71% of serum ucOC variability was explained by these two parameters.

In the entire cohort, the inflammatory marker Hs-CRP correlated with both OC (r = −0.295, p = 0.008) and ucOC (r = −0.236, p = 0.037) but these associations were rendered non-significant after controlling for fat mass.

## Discussion

The study findings indicate that alterations in γ–carboxylation of OC, in connection to weight status and androgen hormones excess may depict PCOS. Participation of serum free testosterone as independent predictor of ucOC, determining about half of ucOC variability when corroborated to bone resorption rate, suggests regulatory interrelationships between androgens and OC metabolism in women, implying potential involvement of the skeleton as an endocrine organ in the pathogenesis of PCOS. In addition to that, the positive association of ucOC with serum insulin observed in lean study participants is a finding which is in agreement to the key effect of ucOC to directly augment pancreatic insulin production and delivery. In turn, within the bone-pancreas regulatory loop, hyperinsulinemia, a feature of PCOS, may promote bone matrix OC decarboxylation and ucOC release *via* the OPG/RANKL pathway [5,21].

Nonetheless, the underlying mechanism linking ucOC to androgen excess in PCOS is less clear. Human visceral adipose cells also express the OC gene, which appears to be up-regulated *in vitro* by dihydrotestosterone, an effect which in men may be reflected by the positive correlation of serum free testosterone with circulating ucOC levels and the ucOC/OC ratio [22]. In male mouse Leydig cells, the ability of osteoblastic ucOC to promote testosterone gene expression at the transcriptional level was recently acknowledged by Oury et al. [23] as an effect accomplished *via* a G-protein coupled orphan receptor which belongs to the C family of GCPRs expressed in mice Leydig cells. Thus, a molecular basis was provided to the positive association between serum OC and free testosterone levels observed in adult men or paralleling skeletal growth in boys of pubertal age [24,25]. However, mouse ovarian follicle cells appear not to express the OC signaling pathway identified in Leydig cells and regulation of sex steroids synthesis in the ovary by ucOC is not yet demonstrated [23]. Notwithstanding, it has to be kept in mind that in the study of Oury et al. testosterone regulation by osteoblast-derived OC was tested in healthy mice ovary explants, an experimental model not superposable to the physiopathological mechanisms governing androgen regulation in PCOS.

Consistent with previous reports [9], both body mass and body fat exerted a strong lowering effect on ucOC, independently of the study subgroup. In fact, obvious weight-dependent relationships of ucOC with several endocrine and metabolic PCOS parameters became apparent in the present study. While ucOC was positively related to both hyperandrogenemia and insulin concentration in lean women, significant associations of ucOC to PCOS traits lacked in the overweight and obese subgroup pointing out to potential implication of additional metabolic players affecting the relationship between ucOC and endocrine parameters in PCOS. There is strong evidence that leptin, one fat tissue-derived adipocytokine, impairs OC production and bioactivity in the skeleton by a central serotonin-mediated activation of the sympathetic nervous system [26]. In mice, leptin up-regulates osteoblastic ESP (*Embryonic Stem cell specific Phosphatase*) gene expression *via* adrenergic β_2_ and ATF4 receptors, an effect associated with accelerated γ-carboxylation of OC, low ucOC and low insulin secretion and sensitivity [27]. Although not assessed in the present study, one may assume hyperleptinemia associated to increased fat mass contributed to the lower OC and ucOC levels observed in overweight and obese participants. In rats fed a high-fat diet, obesity resulted in activation of PPAR (*peroxisome proliferator-activated receptor*)-gamma and suppression of Wnt/β-catenin pathways both associated with stimulation of bone marrow adipogenesis and decreased osteoblast differentiation, as a substrate to diminished OC concentration [28]. Nevertheless, the relationship between OC and fat mass appears to be reciprocal, since a decrease in serum ucOC concentration induced by anti-catabolic bone agents was able to predict long-term accumulation of fat mass [11].

Likewise, in a population-based sample of healthy children, ucOC was related to metabolic parameters in a weight-dependent manner. Higher relative circulating ucOC levels were associated to higher HOMA-IR in leaner but not heavier subjects and to higher high molecular weight (HMW)-adiponectin concentration, an association more apparent in heavier children [29], presumably in order to compensate for the low adiponectin state in heavy subjects. Expression of adiponectin receptors in osteoblasts and regulation of bone metabolism by adiponectin both *in vitro*[30] and *in vivo*[31,32] in addition to increased adiponectin gene expression induced by ucOC could support the concept of an adiponectin-OC loop [3,29] and the increased ucOC delivery in response to hypoadiponectinemia.

As shown here, serum OC assayed as both 1–49 OC and the more stable N-mid (1–43) fragment rather poorly reflects bone-energy metabolism axis status. In part, this may be attributable to factors such as specificity of the assay used, differences in pre-analytical sample stability or large intra-individual variations [33].

As a conclusion, in a weight-dependent manner, the ucOC secretion pattern is related to PCOS status. In lean women, high ucOC levels are strongly predicted by testosterone excess and in turn, may contribute to increased insulin secretion to compensate for altered insulin sensitivity. Future studies are warranted to investigate possible pathogenetic implications of ucOC in the development of PCOS metabolic traits and its potential usefulness as a clinical tool.

## Conclusions

Bone marker under-carboxylated osteocalcin (ucOC) exhibits a weight-dependent pattern in patients with PCOS. Circulating ucOC is predicted by androgen excess in lean women and may stimulate compensatory insulin secretion within the bone-pancreas loop, a mechanism altered by presence of obesity.

## Abbreviations

AE-PCOS: Androgen excess-polycystic ovary syndrome; BMI: Body mass index; cFT: Calculated free testosterone; cOC: Carboxylated osteocalcin; DBP: Diastolic blood pressure; FM: Fat mass; HOMA-IR: Homeostasis model assessment of insulin resistance; Hs-CRP: High-sensitivity C-reactive protein; OC: Osteocalcin; OPG: Osteoprotegerin; PCOS: Polycystic ovary syndrome; QUICKI: Quantitative insulin sensitivity check index; RANKL: Ligand of receptor activator of nuclear factor kappa B; SBP: Systolic blood pressure; SHBG: Sex-hormone binding globulin; TT: Total testosterone; UcOC: Under-carboxylated osteocalcin; WHR: Waist-to-hip ratio.

## Competing interests

The author declares that there is no conflict of interest that would prejudice the impartiality of this scientific work.

## Author’s contribution

CEP was involved in study design, patients’ investigation and manuscript writing.
